# Artificial Intelligence-Assisted Dermatologic Screening: Epidemiology and Clinical Features of Basal Cell Carcinoma, Squamous Cell Carcinoma, Seborrheic Keratosis and Actinic Keratosis

**DOI:** 10.3390/bioengineering12111258

**Published:** 2025-11-17

**Authors:** Teng-Li Lin, Kun-Hua Lee, Riya Karmakar, Arvind Mukundan, Jeevitha Sundarraj, Chun-Te Lu, Shang-Chin Hsieh, Hsiang-Chen Wang

**Affiliations:** 1 Department of Dermatology, Dalin Tzu Chi Hospital, No. 2, Min-Sheng Rd., Dalin Town, Chiayi 62247, Taiwan; tanglilin1121@hotmail.com; 2 Department of Trauma, Changhua Christian Hospital, No. 135, Nanxiao St., Changhua County, Changhua City 50006, Taiwan; 88847@cch.org.tw; 3 Department of Mechanical Engineering, National Chung Cheng University, 168, University Rd., Min Hsiung, Chia Yi 62102, Taiwan; karmakarriya345@gmail.com (R.K.); arvindmukund96@gmail.com (A.M.); 4 School of Engineering and Technology, Sanjivani, University, Sanjivani Factory, Singnapur, Kopargaon 423603, Maharashtra, India; 5 Department of Biomedical Imaging, Chennai Institute of Technology, Sarathy Nagar, Chennai 600069, Tamil Nadu, India; 6 Department of Biotechnology, Karpagam Academy of Higher Education, Salem-Kochi Hwy, Eachanari, Coimbatore 641021, Tamil Nadu, India; jeevithasundarraj12@gmail.com; 7 Institute of Medicine, School of Medicine, College of Medicine, National Yang Ming Chiao Tung University, No. 155, Sec. 2, Li-Nong Street, Beitou District, Taipei 112304, Taiwan; 8 Department of Surgery, Division of Plastic and Reconstructive Surgery, Taichung Veterans General Hospital, 1650 Taiwan Boulevard Sect. 4, Taichung 407219, Taiwan; 9 Department of General Surgery, Kaohsiung Armed Forces General Hospital, 2, Zhongzheng 1st Rd., Lingya District, Kaohsiung City 80284, Taiwan; 10 Department of Medical Research, Dalin Tzu Chi Hospital, Buddhist Tzu Chi Medical Foundation, No. 2, Minsheng Road, Dalin, Chiayi 62247, Taiwan; 11 Technology Development, Hitspectra Intelligent Technology Co., Ltd., Kaohsiung 80661, Taiwan

**Keywords:** skin cancer, support vector machines, convolutional neural networks, squamous cell carcinoma, seborrheic keratosis, actinic keratosis

## Abstract

This literature review synthesizes contemporary evidence regarding the epidemiology, screening guidelines, clinical manifestations, and machine-learning solutions for four prevalent non-melanoma skin lesions: basal cell carcinoma (BCC), squamous cell carcinoma (SCC), seborrheic keratosis (SK), and actinic keratosis (AK). This study presents a summary of common indices and recent screening alternatives, accompanied by a critical assessment of contemporary advancements in artificial intelligence (AI) and machine learning (ML) for the identification and classification of images utilizing standardized benchmark databases. The literature search and selection focused on peer-reviewed studies published from 2018 to December 2024, emphasizing diagnostic performance, datasets, preprocessing methodologies, and assessment metrics. This work compares and contextualizes reported results, highlighting the challenges posed by different study designs and biases in datasets that hinder direct comparisons among studies. The consistency of deep learning classifiers in lesion detection, the significance of sensitivity-oriented thresholding for early detection applications, and challenges associated with class imbalance and the under-representation of darker skin tones in publicly accessible datasets are studied. With practical implications for clinical adoption, emphasizing targeted screening of at-risk populations, the supplementary benefits of dermoscopy and the imperative for multi-center, demographically diverse validation have been concluded. Additionally, future research on standardized reporting, external validation, and interpretable, workflow-compatible AI systems has been proposed.

## 1. Introduction

Skin cancer is defined by the existence of atypical cells and is classified into two main types: melanoma and non-melanoma [1,2]. Non-melanoma skin cancer (NMSC), while less common than other types, has presented a considerable concern to those with fair skin worldwide for several decades [3]. Melanoma is a common type of skin cancer that accounts for several fatalities associated with skin cancer [4]. The incidence of melanoma surpasses that of non-melanoma skin malignancies due to heightened cumulative ultraviolet (UV) exposure. The NMSC exposure amplifies the sun-seeking tendencies of UV light and ozone depletion [5]. The NMSC exposure exacerbates the sun-attracting properties of UV radiation and the depletion of ozone. The prevalence of non-melanoma skin cancer (NMSC) is reported to be as high as 2398 per 100,000 individuals in Australia, around 232 per 100,000 in the United States, and 1908 per 100,000 for both sexes combined. [6]. Non-melanoma skin cancers (NMSCs) consist of two principal types: basal cell carcinoma (BCC) and squamous cell carcinoma (SCC) [7]. In the United States, basal cell carcinoma (BCC) is the most common type of skin cancer, distinguished by a painless raised area of skin that may exhibit a shiny look with ulceration [8,9]. Ultraviolet exposure, radiation therapy, and impaired immune function are hazardous risks for basal cell carcinoma (BCC) [10]. BCC includes multiple subtypes, including nodular, superficial, and morpheaform BCC [11]. Nodular BCC (nBCC) typically manifests as an erythematous, dome-shaped nodule, predominantly located on the neck and head [12]. The risk factors for BCC include ultraviolet (UV) exposure and immunosuppression [13]. Superficial basal cell carcinoma (sBCC), along with nodular and aggressive variants, appears on sun-exposed skin in elderly individuals [14]. Morpheaform basal cell carcinoma (MorBCC) is an aggressive variation of basal cell carcinoma that primarily occurs on the face and neck [15].

Squamous cell cancer (SCC) arises from epidermal keratinocytes due to factors like prolonged sun exposure, advanced age, immunosuppression, and pale skin [16]. Squamous cell carcinoma (SCC) can present as various lesions, such as scaly red patches, open sores, thickened wart-like growths, or raised nodules. Squamous cell carcinoma (SCC) typically develops due to ultraviolet radiation exposure, with around one-third of SCC lesions classified as Bowen’s disease (BD) or noninvasive [17,18]. Radiologic imaging, including computed tomography (CT), magnetic resonance imaging (MRI), positron emission tomography-computed tomography (PET-CT), and ultrasonography of the regional lymph nodes, is advised for patients with high-risk cutaneous squamous cell carcinoma (cSCC) [19]. Seborrheic keratosis (SK) is a prevalent benign dermal lesion distinguished by a waxy, adhering brown or black appearance and a rough texture, frequently found on the face, chest, shoulders, and back [20,21]. SK measures a few to several millimeters and presents as a roundish, reddish to brownish scaling lesion [22]. Seborrheic keratosis is a benign proliferation of skin cells predominantly observed in elderly persons. Diagnosis typically relies on clinical manifestations, including milia-like cysts, comedo-like holes, and fissures or ridges. A biopsy is performed solely on lesions that are atypical or evolving and arouse suspicions of melanoma, pigmented basal cell carcinoma, or other dermatological conditions that may resemble them. The differential diagnosis must explicitly encompass solar lentigo, verruca vulgaris, lichenoid keratosis, and melanoma. Lesions that lack a definitive diagnosis or exhibit new symptoms such as hemorrhage, accelerated progression, or ulceration require confirmation through histopathological examination. Management is often unwarranted unless lesions are symptomatic, injured, or esthetically displeasing. Typical office-based interventions encompass cryotherapy, curettage or shave excision with or without electrodessication, and specific laser or energy techniques. The choice of treatment is determined by the size, location, pigmentation of the lesion, and the preferences of the patient. A recent dermatological review consolidates novel insights about seborrheic keratoses in terms of epidemiology, pathobiology, and treatments. It discusses the characteristic clinical and dermoscopic patterns, their propensity for spontaneous resolution, and the significance of distinguishing between seborrheic keratosis and malignant mimics in routine therapy [23]. The review consolidates information for destructive and ablative methods and examines patient-reported results, esthetic factors, and adverse effect profiles to facilitate shared decision-making. These sources collectively provide a concise, dermatology-focused summary of the pathogenesis, diagnosis, and management of seborrheic keratosis, ensuring that this section of the publication aligns with contemporary clinical practice [24].

Hyperkeratosis is characterized by the production of blisters, aggregation of keratin, and degradation of skin cells [25]. Acanthosis is characterized by dark-brown hyperpigmentation and a velvety thickening of the skin, commonly occurring in the neck, groin, and other flexural areas [26]. Human papillomavirus (HPV) affects children and young adults, resulting in papillomatous lesions in the aerodigestive tract [27].

Actinic keratosis (AK) is a prevalent dermatological condition resulting from prolonged sun exposure, with 75% of cases manifesting in areas frequently exposed to sunlight, including the hands, forearms, neck, scalp, and face [28]. Extended sun exposure caused unusual changes in the epidermal keratinocytes attributed to actinic keratosis [29]. AK presents as an indistinct papule or plaque, displaying a color range from skin-toned to pink, red, or brown, along with dry, scaly lesions [30]. AK involves processes such as oxidative stress, immunosuppression, inflammation, changes in cell growth and proliferation, inhibited apoptosis, mutagenesis, and HPV [31]. There are two categories of therapy for actinic keratosis: lesion-directed therapy and field-directed therapy. Cryoablation, curettage, or Neosporin are applied directly to the lesion [32].

Seborrheic keratosis is a benign growth of skin cells primarily seen in older individuals. Diagnosis typically depends on clinical presentations, including milia-like cysts, comedo-like openings, and fissures or ridges. A biopsy is conducted solely on unusual or developing lesions that raise suspicions of melanoma, pigmented basal cell carcinoma, or other dermatological disorders that may mimic them. The differential diagnosis must specifically include solar lentigo, verruca vulgaris, lichenoid keratosis, and melanoma. Lesions without a specific diagnosis or presenting novel symptoms (such as hemorrhage, rapid advancement, or ulceration) demand proof via histopathological testing. Management is often needed unless lesions are symptomatic, damaged, or cosmetically unappealing. Standard office-based therapies include cryotherapy, curettage, shave excision (with or without electrodessication), and targeted laser or energy technologies. The selection of treatment is influenced by the size, location, coloration of the lesion, and the patient’s preferences.

A recent dermatological review synthesizes novel findings about seborrheic keratoses, focusing on epidemiology, pathobiology, and therapeutic options. It examines the distinctive clinical and dermoscopic patterns, their likelihood of spontaneous remission, and the importance of differentiating between seborrheic keratosis and malignant mimics in standard treatment. The assessment synthesizes data on destructive and ablative techniques (e.g., cryotherapy, curettage/shave, electrosurgery, and laser operations) and evaluates patient-reported outcomes, esthetic considerations, and adverse effect profiles to enhance shared decision-making. These sources jointly offer a succinct, dermatology-centric overview of the pathological process, diagnosis, and treatment of seborrheic keratosis, ensuring that this section of the article conforms to current clinical standards.

This research investigation offers a comprehensive examination of the epidemiology, screening, and clinical features of four common non-melanoma skin lesions: BCC, SCC, SK, and AK, as well as a critical evaluation of current developments in AI and ML pertinent to the diagnosis of these lesions. This study will explicitly evaluate the diagnostic efficacy of deep learning and machine learning models over three benchmark dermatological datasets, namely ISIC and HAM10000. The main goal is to emphasize recent progress in AI-based diagnostic methods, advocate for their clinical use, and underscore their promise for early, accurate, and non-invasive identification of skin cancer. The literature review encompassed peer-reviewed papers published from 2018 to December 2024. Articles published after this period were excluded, as the data gathering and analysis steps had been finished before the release of research in 2025. Nevertheless, the chosen works embody the most current and impactful developments at the time of the evaluation.

## 2. Clinical Features

BCC represents the most predominant type of human malignancy globally, comprising roughly 70 to 80% of all non-melanoma skin malignancies. The prevalence of the disorder is rising worldwide, especially among those with light skin and in regions with elevated UV radiation exposure; yet, its fatality rate remains low due to its indolent character and high treatability with timely intervention. Squamous cell carcinoma (SCC) ranks as the second most common keratinocyte carcinoma, accounting for roughly 20–25% of non-melanoma lesions, with a metastasis risk of 1–5%, especially in immunocompromised or overlooked instances. SK and AK are benign or pre-malignant lesions that are important markers of chronic UV exposure and act as effective indications for identifying at-risk groups. Recent reviews in dermatologic epidemiology literature support these increasing trends and highlight the necessity for improved preventative efforts focusing on sun protection and early diagnosis. Concerning screening, it is essential to acknowledge that prominent guideline organizations currently do not advocate for routine visual skin assessments for asymptomatic individuals. The U.S. Preventive Services Task Force (USPSTF, 2023) concluded that there is insufficient evidence to endorse or oppose routine screening for skin and related cancers [33]. Consequently, dermatological screening predominantly focuses on high-risk populations, such as persons with fair skin, extensive sun exposure, more than 100 nevi, or a history of skin malignancies, rather than general population efforts. Thus, photo-protection, patient education, and routine self-examination represent the most effective preventive measures [34]. Clarifying this distinction ensures the review adheres to current clinical guidelines and mitigates the risk of misinterpreting the screening recommendations.

### 2.1. Nodular Basal Cell Carcinoma (nBCC)

**Figure 1:**
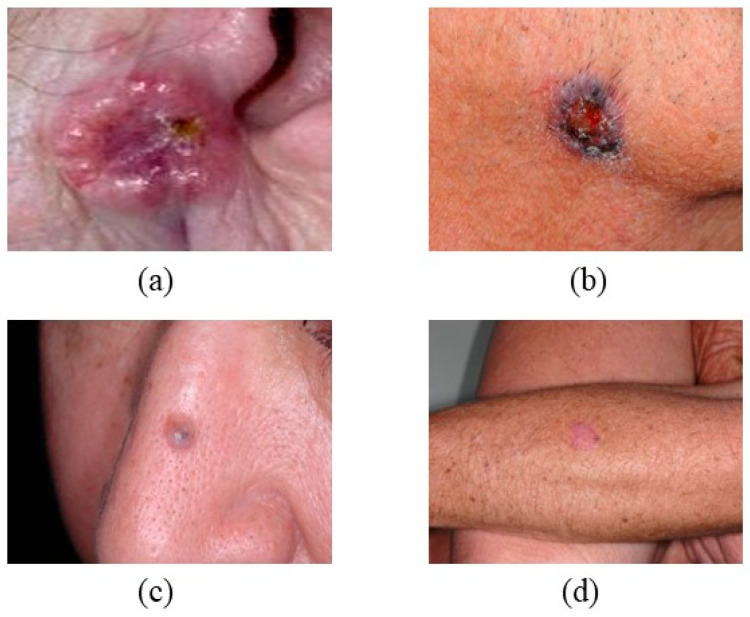
Shows that nBCC has different locations and different Colors (**a**) a red, pearly spot on the ear, (**b**) brown-black discoloration on the neck, (**c**) a pearly bump on the nose, and (**d**) a pink growth on the leg. Basal cell carcinoma (BCC) on the nasal bridge: note the pearly rolled border, central ulceration, and telangiectatic vessels characteristic of nodular BCC. The lesion’s translucency and shiny surface are typical of slow-growing keratinocyte carcinomas. (Image source: ISIC Archive.).

nBCC constitutes 75% of all BCC cases and frequently presents in superficial or ulcerated regions on sun-damaged skin [35]. nBCC a (BCC) appears on the face and neck, characterized by endophytic nodules that often emerge as flat, persistent plaques, as depicted in Figure 1. These nodules may ulcerate, contain well-defined edges, reveal thickness upon probing, display a yellowish-white tint, and feature a flat surface area [36,37]. Nodular basal cell carcinomas measuring less than 0.15 cm in diameter constitute almost 50% of all BCC instances [38]. The nodules may have differences in pigmentation and mimic cysts owing to their transparent coloration and flexible feel [39].

The micronodular subtype comprises small, spherical tumor nodules, while the nodular tumor typically attains the size of hair bulbs [40]. Contributing variables may include exposure to chemical carcinogens, potential HPV infection, and ionizing radiation [41]. Red blood cells, gene enrichment in specific categories, cellular proliferation, chromosome segregation, and regulation of cellular growth were identified, among other factors.

### 2.2. Superficial Basal Cell Carcinoma (sBCC)

**Figure 2:**
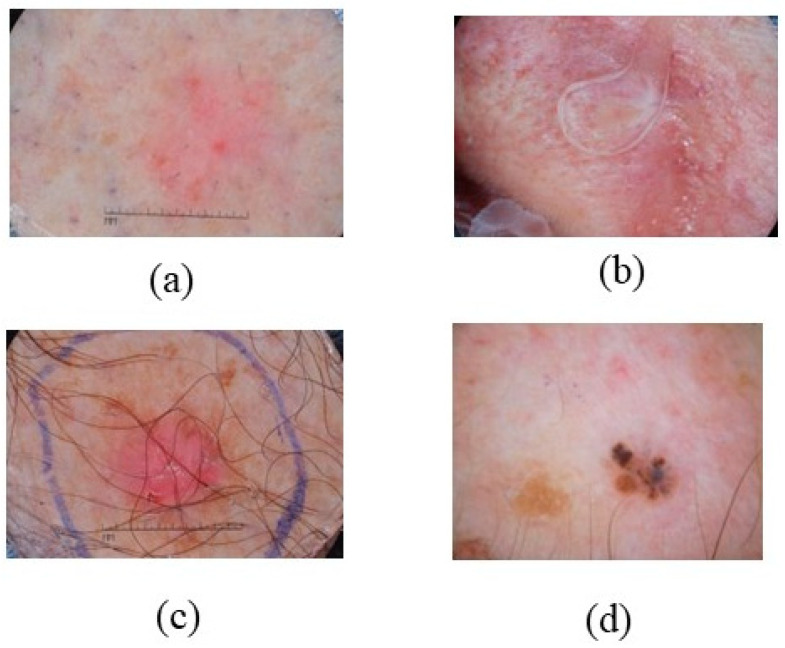
Shows sBCC that location, color, and appearance (**a**) dotted vessels on the leg, (**b**) shiny white to pink on the face, (**c**) scaly pink-red patch on the chest and back, and (**d**) yellow scales on the trunk and extremities. Superficial basal cell carcinoma on the trunk: thin, erythematous plaque with fine rolled margins and focal crusting. Dermoscopic view shows arborizing vessels and map-like pigmentation. (Image source: ISIC Archive.).

Topical imiquimod is an effective noninvasive therapy for superficial basal cell carcinoma, presenting as pink-red scaly patches or macules. Lesions frequently manifest on the trunk and extremities; therapeutic options encompass electrodesiccation and curettage, cryotherapy, and topical chemotherapeutics [42,43]. It affects areas with considerable sun exposure, such as the head, face, legs, chest, back, and neck, with sBCC being particularly effective on flat surfaces and several minor erosions, as depicted in Figure 2 [44,45]. Basal cell carcinoma predominantly affects younger adults and females, particularly in areas with substantial sun exposure. Superficial basal cell carcinoma (sBCC) can be treated with imiquimod, fluorouracil cream, photodynamic therapy, and cryotherapy [46].

### 2.3. Morpheaform Basal Cell Carcinoma (MorBCC)

**Figure 3:**
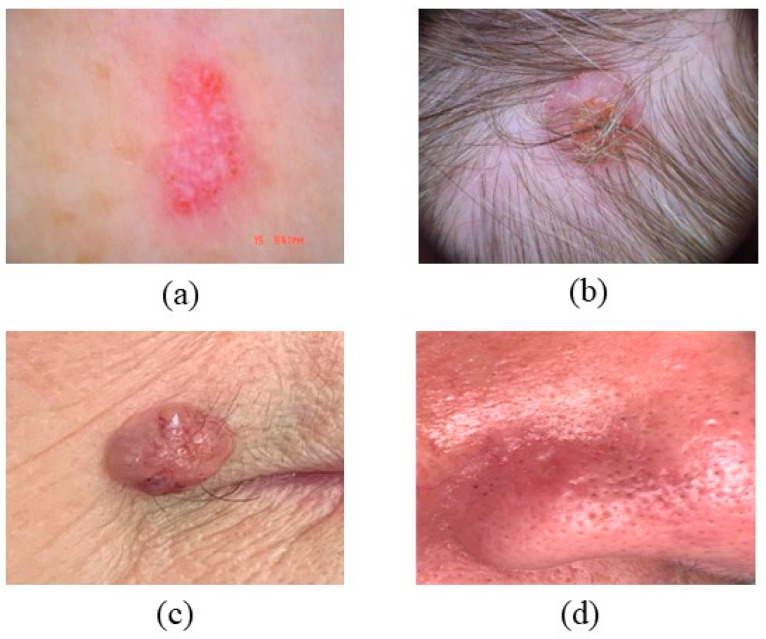
Shows MorBcc that location and appearance, which arises in the scar (**a**). It has a waxy and ulcerated appearance on the scalp (**b**). It is in the pearly form in the eyelid (**c**). The reddish patch is located in the nose area (**d**). Superficial basal cell carcinoma on the trunk: thin, erythematous plaque with fine rolled margins and focal crusting. Dermoscopic view shows arborizing vessels and map-like pigmentation. (Image source: ISIC Archive.).

**Figure 4:**
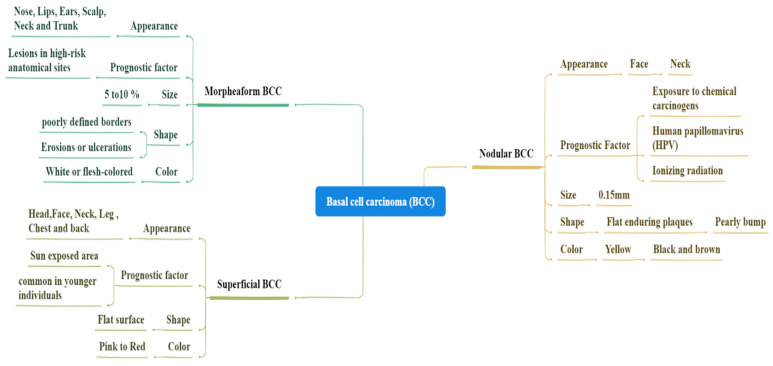
Illustrates the histopathological and clinical diversity of BCC subtypes, emphasizing differences in growth pattern, pigmentation, and stromal interaction. The figure highlights the structural variations between nodular, superficial, and morpheaform subtypes, showing how these differ in border definition, infiltration depth, and pigmentation.

MorBCC is a subtype of BCC, the most prevalent skin disease, and constitutes an aggressive histological variant of the most commonly diagnosed skin cancer [47,48]. Mor-BCC typically presents as a smooth plaque that is either white or flesh-toned, featuring firm areas and indistinct margins, and may have erosions or ulcerations inside a sclerotic plaque, as depicted in Figure 3 [49]. MorBCC typically manifests on the face, scalp, nose, and neck [50]. Approximately 5% to 10% of BCCs manifest as the morphea or sclerosing variant, as illustrated in Figure 4 [51]. The five-year recurrence rate for morpheaform basal cell carcinoma (morBCC) is 16.3%, increasing when lesions are located in high-risk regions such as the nose, lips, ears, head, trunk, and neck [52].

### 2.4. Squamous Cell Carcinoma (SCC)

**Figure 5:**
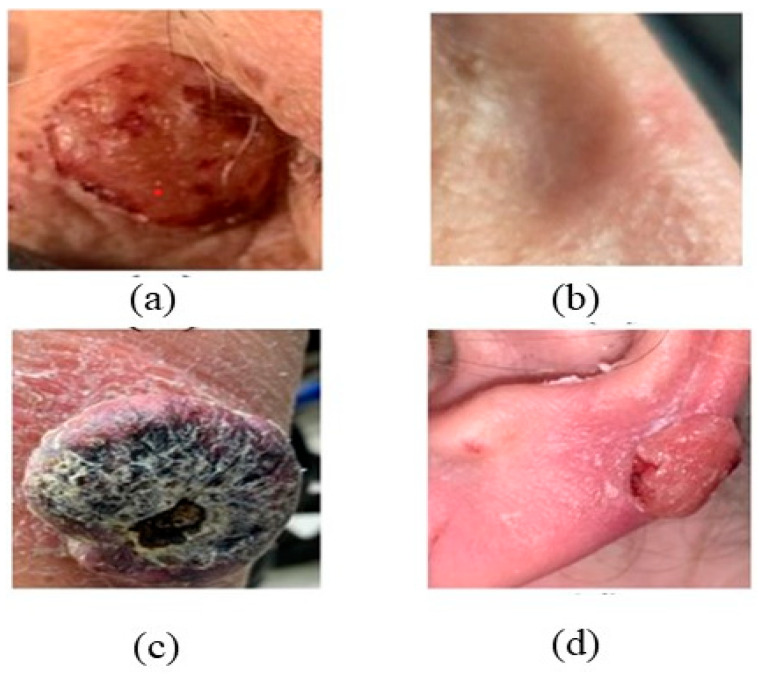
Presents representative clinical appearances of squamous cell carcinoma (SCC) across various anatomical regions. (**a**) Ulcerated SCC presenting as a raised erythematous nodule with an eroded surface and indurated border; (**b**) Ill-defined erythematous, slightly scaly plaque on the nasal region, representing a superficial / early SCC lesion; (**c**) Keratoacanthoma-type SCC on the forearm, showing a dome-shaped nodule with a central keratin-filled crater and thick crusted, wart-like surface; these lesions grow rapidly and may regress spontaneously; (**d**) Exophytic nodular SCC of the ear with surface scale and focal ulceration, appearing clinically as an open sore on the auricular margin. The lesions exhibit a spectrum of morphologies, including ulcerated nodules, erythematous scaly plaques, open sores, and crusted wart-like growths. These visual differences correspond to varying stages of differentiation and invasion within SCC progression. Keratoacanthoma-type SCC on the forearm: dome-shaped nodule with a central keratin plug and erythematous surrounding rim. Such lesions demonstrate rapid growth but may regress spontaneously. (Image source: ISIC Archive.).

**Figure 6:**
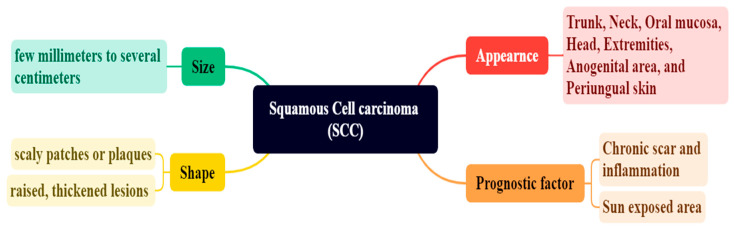
Characteristics of SCC.

Squamous cell carcinoma (SCC) may occur in several locations, including the trunk, neck, oral mucosa, head, extremities, anogenital region, and periungual skin [53]. In situ squamous cell carcinoma (SCC) is a type of skin cancer primarily impacting the epidermis of basal cell carcinoma (BCC) and frequently manifests in adults over the age of 60. The morphology of BD differs based on the site of origin, lesion age, and degree of keratinization, as depicted in Figure 5 [54,55]. BD presents as a clearly defined, erythematous, progressively increasing, scaly region or plaque, as depicted in Figure 5 [56]. The tumor may range in size from a few millimeters to several centimeters, contingent upon the duration of the disease, as depicted in Figure 6 [57]. Minor symptoms, such as pruritus or burning sensations, may occur, and those with fair skin over the age of 50 often acquire a prevalent lesion referred to as keratoacanthoma. The risk factor of SCC causes enduring scarring and chronic inflammation [58,59].

### 2.5. Seborrheic Keratosis (SK)

**Figure 7:**
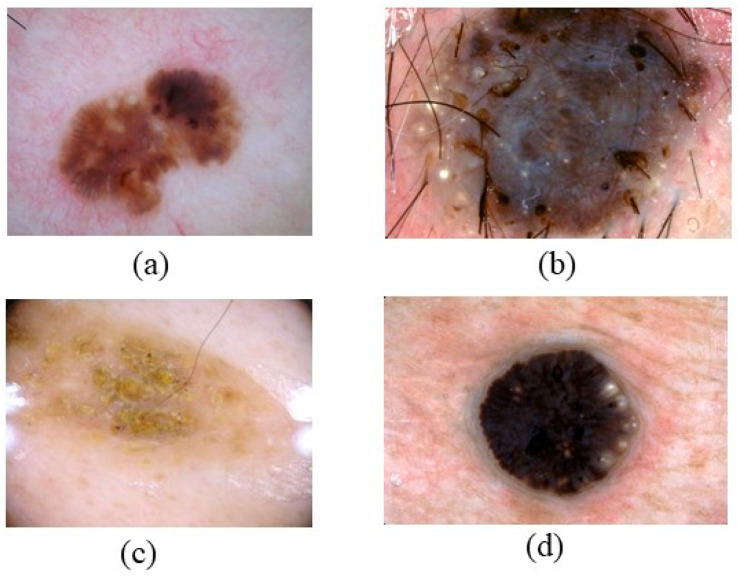
Shows SK that color and location (**a**) brown, black, or yellow, (**b**) resembling warts or moles, (**c**) found on the scalp, face, chest, or back, and (**d**) varying in texture. Seborrheic keratosis (SK) on the upper back: sharply demarcated, waxy “stuck-on” appearance with milia-like cysts and comedo-like openings visible under dermoscopy. These benign lesions often mimic pigmented BCC or melanoma. (Image source: ISIC Archive.).

**Figure 8:**
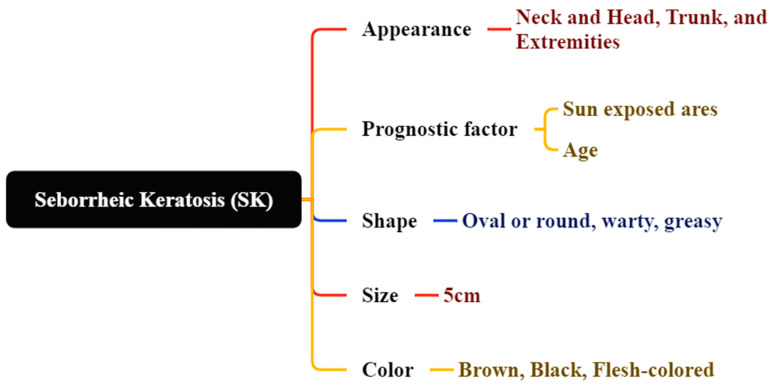
Properties and Characteristics of SK.

SK is the most prevalent benign epidermal neoplasm, impacting both sexes equally [60]. SK manifests as oval or circular, brown-black warty plaques with a greasy texture or flesh-colored appearance [61]. SK may present on the neck, head, trunk, and extremities, especially in regions with hair-bearing skin [62]. It generally manifests in individuals aged 61 to 85 years, appears on sun-exposed skin regions, and presents as well-defined, slightly elevated brownish patches or plaques, as depicted in Figure 7 [63,64]. Subtypes of SK generally display three features: acanthosis, hyperkeratosis, and papillomatosis. Diagnosing pigmented seborrheic keratosis necessitates the assessment of conditions including blue nevus, melanocytic nevus, solar lentigo, pigmented basal cell carcinoma, and pigmented actinic keratosis, as seen in Figure 8 [65]. Exophytic, hyperplastic, and hyperpigmented changes may also occur in seborrheic keratosis, with lesions possibly reaching a diameter of 5 cm [66,67].

### 2.6. Actinic Keratosis (AK)

**Figure 9:**
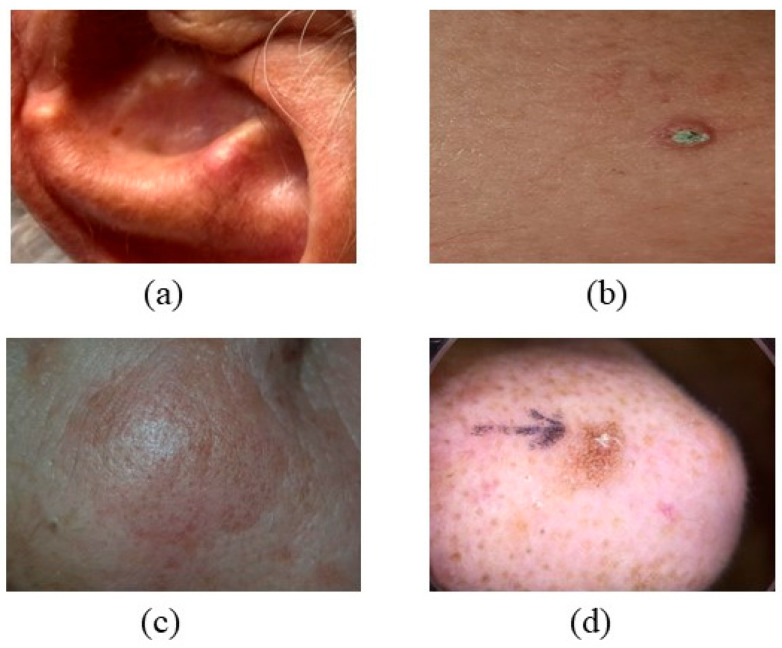
Shows AK’s location, color, and appearance (**a**) located on the ear, face, back of hands, and neck, (**b**) colored white or yellow, (**c**) with a scaly and wart-like appearance, and (**d**) often presents as raised lesions. Actinic keratosis (AK) on the forehead: rough, erythematous scaly plaque with surrounding photodamaged skin. Dermoscopy shows white-to-yellow scales and red pseudonetwork, indicating early keratinocytic atypia. Multiple actinic keratoses on sun-exposed scalp: confluent hyperkeratotic papules and plaques on atrophic skin. The image highlights field cancerization in chronically UV-damaged areas, emphasizing the importance of preventive photoprotection. (Image source: ISIC Archive.).

**Figure 10:**
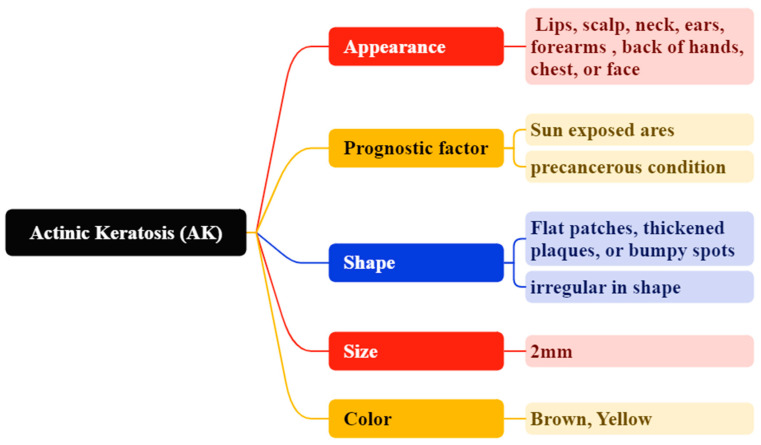
Properties and Characteristics of AK.

Actinic keratosis (AK) is a precancerous condition caused by sun exposure, presenting as small nodules, flat lesions, or larger plaques [68]. Actinic keratosis (AK) presents as thick, scaly, or crusty lesions that are often dry or rough, commonly occurring in sun-exposed regions such as the lips, scalp, neck, ears, forearms, dorsal hands, chest, or face [69]. The lesions typically exhibit yellow or brown scales and have a diameter of approximately 2 mm, as depicted in Figure 9 [70,71]. Individuals with fair skin and the elderly demonstrate an increased risk. The mechanisms involved in AK include inflammation, impaired apoptosis, oxidative stress, cell cycle dysregulation, increased cell proliferation, immunosuppression, and changes in tissue architecture, as illustrated in Figure 10 [72]. Lesions associated with actinic keratosis (AK) exhibit visible damage, including solar elastosis, dyspigmentation, ephelides, yellow discolouration, lentigos, telangiectasias, and sagging skin, with around 50–77% of lesions extending to a depth of 300 micrometers within the AK [73,74].

## 3. Screening and Diagnosis of BCC, SCC, SK, and AK

### 3.1. Screening and Diagnosis of BCC

Pathophysiology: BCC arises from basal cells located beneath the epidermis, including hair follicles and eccrine sweat ducts [75]. Modifications in the Hippo pathway are associated with the advancement of human cancer by causing anomalies in tissue growth patterns [76]. Ultraviolet radiation (UVR) is the principal etiological factor for BCC [77]. BCCs generally display branching blood vessels, ulceration, and blue-gray ovoid nests, indicating changes in the tumor’s vascular structure and cellular composition [78].

Etiology: Ultraviolet radiation causes DNA damage and immunosuppression, promoting the development of basal cell carcinoma (BCC). It may diminish the quantity of antigen-presenting cells [79]. Chronic UVR exposure, especially UVB, constitutes the principal risk factor [80]. While the risk factors of chronic UVR, particularly UVB are the primary risk factor [81]. Extended exposure leads to keratinocyte carcinogenesis, resulting in basal cell carcinoma (BCC) after years or decades of cumulative cellular damage [82].

Evaluation: Assessment: The pathology report must specify the histologic subtype of basal cell carcinoma (BCC), the degree of tumor penetration beyond the reticular dermis, and any perineural invasion [83].

Treatment: Common methods for excising basal cell carcinoma (BCC) encompass radiation, curettage, topical chemotherapy (5-fluorouracil and imiquimod), electro-desiccation, conventional surgical excision, and Mohs micrographic surgery (MMS) [84]. Mohs surgery for basal cell carcinoma (BCC) is the most efficacious method for completely excising the tumor by microscopically analyzing all margins of the excised tissue and resecting extra tissue if cancer cells are identified [85,86,87]. The primary forms of radiation therapy consist of superficial or orthovoltage radiation therapy, electron beam therapy, and high-dose-rate brachytherapy [88].

### 3.2. Screening and Diagnosis of SCC

Pathophysiology: A 68-year-old female smoker received a diagnosis of carcinoma in situ on the left anterior ventral aspect of the tongue. The excisional biopsy margins displayed considerable epithelial dysplasia post-procedure [89]. cSCC genetically arises from keratinocytes, the predominant cell type in the epidermis, which undergo malignant transformation. The predominant cause of this alteration is extended exposure to UV radiation, which directly damages DNA. The mutation transpires in the TP53 tumor suppressor gene, the most common genetic alteration in cSCC caused by UVB, resulting from a cytosine-to-thymine transition. Further mutations in tumor suppressor genes (CDKN2A and NOTCH1/2), in addition to infrequent modifications in oncogenes such HRAS [90]. The risk factors for cutaneous squamous cell carcinoma (cSCC) closely resemble those of other squamous cell carcinomas (SCCs), primarily encompassing frequent exposure to solar ultraviolet radiation (UVR) and ionizing radiation, chronic immunosuppression in organ transplant recipients, and human papillomavirus (HPV) infection [91]. The principal risk factor is extended exposure to ultraviolet radiation, commonly associated with lesions located on sun-exposed regions of the head and neck [92]. Ionizing radiation and HPV infections are major predisposing variables in the etiology of cSCC. Patients are at considerable risk of developing aggressive cancer forms, especially those with a history of transplantation and extended immunosuppression [93].

Etiology: Ultraviolet radiation (UVR) is the principal risk factor for the development of squamous cell carcinoma (SCC), with mutations in the TP53 gene frequently linked to UVR-induced DNA damage [41,94]. Chemicals, particularly pesticides and industrial substances such as polycyclic aromatic hydrocarbons (PAHs) and arsenic, increase the risk of cancer. These substances may facilitate tumor proliferation, disturb hormonal balance, and compromise the immune system [95].

Epidemiology: The incidence of squamous cell carcinoma (SCC) is significantly higher in transplant recipients compared to the general population, with ultraviolet radiation (UVR) recognized as the primary causative factor for cutaneous cancer in both groups. In Australia, up to 70% of transplant recipients develop squamous cell carcinoma (SCC) within 20 years following transplantation. In transplant recipients, squamous cell carcinoma (SCC) generally demonstrates rapid proliferation and results in elevated mortality rates [96].

Treatment: Mohs surgery for squamous cell carcinoma (SCC) removes the tumor in stages with concurrent microscopic analysis, resulting in lower recurrence rates and faster recovery times. It requires specialized expertise and may be expensive. Conventional excision involves the extraction of malignant tissue, subsequently requiring a period for conclusive histological assessment, which may lead to extended wait periods, elevated recurrence rates, and the need for additional surgical procedures [97]. Two principal modalities of radiation therapy utilized in the management of skin cancer are external beam radiation therapy and brachytherapy (BT) [98]. External beam radiation therapy is employed to target and treat internal cancerous lesions [99]. Brachytherapy (BT) is a form of radiation that employs a radioactive source positioned within or next to the tumor [100]. Superficial and interstitial brachytherapy are the two techniques utilized for the treatment of skin cancer. A superficial brachytherapy radiation source is placed near the skin, whereas interstitial brachytherapy radioactive isotopes are directly embedded within the tumor for deeper intervention [101]. Cryotherapy, also referred to as cryosurgery, is a frequently utilized intervention for squamous cell carcinoma (SCC) and numerous other dermatological conditions. Cryotherapy utilizes extreme cold to eradicate cancer cells. The method is expeditious, does not necessitate anesthesia, and can treat many lesions in one session. Cryotherapy is a primary treatment for lentigo maligna and Bowen’s disease [102].

The radiation and surgical protocols must adhere to the dermatology-specific recommendations. The American Society for Radiation Oncology (ASTRO) clinical practice guideline recommends definitive radiation therapy for non-melanoma skin cancer patients who are unsuitable for surgery or decline surgical intervention. Additionally, adjuvant radiation is advised post-surgery for patients exhibiting particular unfavorable traits (positive or close margins not suitable for further excision, extensive perineural invasion, or regional nodal disease), with the treatment modality and fractionation tailored to the specific site [103]. The dermatology society guidelines delineate first-line management strategies, emphasizing surgical excision and Mohs micrographic surgery (MMS). The British Association of Dermatologists (BAD) advocates for MMS or margin-controlled excision in high-risk basal cell carcinoma, particularly in critical anatomical locations, aggressive histological subtypes, and recurrent tumors, as well as in selected cSCC exhibiting high-risk characteristics such as poor differentiation and perineural invasion [104]. The decision-making process regarding MMS is further structured by the American Academy of Dermatology Appropriate Use Criteria, which categorizes tumor and patient factors by anatomical risk zones and provides relevant scenario ratings and clinical algorithms for practical application [105]. The AAD guidelines for cSCC encompass biopsy, staging, selection of first-line treatment, and indications for adjuvant radiation in high-risk cases, aligning dermatological practice with oncological principles and emphasizing cutaneous considerations. All these sources will provide a dermatology-specific framework for determining when MSS should be selected or when radiation should be employed, either definitively or post-surgery. General oncology citations will be substituted with the aforementioned dermatological and radiation-specific guidelines, thereby rectifying the identified discrepancies in references noted by the reviewer and enhancing methodological consistency.

### 3.3. Screening and Diagnosis of SK

Pathophysiology: The activation of mutations in the FGFR3 gene is crucial to the pathogenesis of SK, which includes various benign skin disorders; dermatosis papulosa nigra (DPN) is characterized by benign, black papules arising from hyperpigmented keratinocytes in individuals with darker skin tones. Stucco keratosis is a benign, wart-like lesion on the lower extremities caused by abnormal skin cell growth, often linked to age and sun exposure. Inverted follicular keratosis (IFK) manifests as benign lesions originating from hair follicle cells and may mimic squamous cell carcinoma (SCC). Lichenoid keratosis (LK) presents as slightly raised, red-brown plaques due to keratinocyte proliferation and lymphocyte infiltration, perhaps resembling basal cell carcinoma (BCC). Flat seborrheic keratoses present as benign tan-brown lesions arising from the growth of skin cells, usually found in sun-exposed areas [106,107].

Epidemiology: The study included 138 patients with clinical diagnoses of seborrheic keratosis (SK), all of whom underwent dermoscopy and skin biopsy for accurate diagnosis. The patients’ ages varied from 24 to 89 years, with an average age of 55.18 years [108].

Etiology: Seborrheic keratosis (SK) is primarily affected by ultraviolet (UV) light exposure, with persons of Fitzpatrick skin types I and II demonstrating more photoaging compared to those with skin types III and IV [109,110]. SKs often appear in locations usually covered by clothing, as well as in the perigenital and intertriginous regions. SKs are predominantly observed in atrophic photoaging rather than in hypertrophic photoaging [111].

Treatment: Conventional remedies for SK include cryotherapy and curettage. Currently, potential topical treatments comprise 40% hydrogen peroxide and a nitric-zinc amalgamation. Ablative laser therapy serves as an alternate treatment modality for seborrheic keratosis [23].

### 3.4. Screening and Diagnosis of AK

Pathophysiology: AK activates several pathways, primarily involving inflammation, oxidative stress, immunosuppression, alterations in apoptosis, mutagenesis, changes in cellular growth and differentiation, and tissue remodeling [112]. AK lesions are generally recognized via visual examination during standard dermatological evaluations. Sunlight exposure results in the development of rough, scaly patches on the skin [113]. Extended UV exposure causes considerable skin damage, a process referred to as field cancerization, affecting extensive areas of the skin [114].

Epidemiology: Despite its considerable prevalence, AK is often underdiagnosed. A survey of physicians indicated that primary care physicians (PCPs) had more familiarity with basal cell carcinoma (BCC) than with actinic keratosis (AK). AK treatment rates shown significant heterogeneity by country, with Europe displaying lower rates in comparison to the US and Australia [115]. Histology assists in differentiating AK from similar conditions, such as SCC and BCC. A biopsy may be performed on suspected lesions to confirm the diagnosis. Histopathological examination reveals epidermal dysplasia, a characteristic feature of actinic keratosis (AK).

Treatment: Actinic keratosis (AK) is addressed with methods including curettage, cryotherapy, and topical medicines such as 5-fluorouracil (5-FU) and diclofenac gel as standard therapies [116]. Imiquimod (IQ) and photodynamic therapy (PDT) are utilized for more complex instances, with PDT producing enhanced esthetic results. In resource-limited environments, economical treatments like diclofenac and retinoids are commonly utilized. Treatment depends on the lesion’s severity, any side effects, and related expenses [117].

## 4. Machine Learning Applications in Skin Cancer Detection

This review mostly analyzed ML and DL studies employing benchmark datasets such as the ISIC Archive and HAM10000, encompassing over 70,000 and 10,015 dermoscopic images, respectively, including representative samples of BCC, SCC, SK, and AK [118,119]. The sample sizes in the analyzed studies varied, with smaller experimental subsets often comprising 500 to 5000 images per lesion class, contingent upon the model architecture and preprocessing methodology.

### 4.1. Basal Cell Carcinoma

BCC is the most prevalent form of human cancer, arising from the basal keratinocytes of the epidermis and hair follicles. It is closely linked to prolonged exposure to UV radiation and usually affects sun-exposed skin, particularly the nose, ears, and neck of those with fair complexions. Nodular basal cell carcinoma is the primary subtype, distinguished by a shiny, pearlescent pink papule or nodule exhibiting minute telangiectatic capillaries on its surface and undulating, translucent margins. With advancing age, the central area of the depression or ulceration may become more pronounced, resulting in the distinctive appearance of a rodent ulcer. The lesion exhibits a smooth surface, characterized by regularly intersecting arborizing vessels. Superficial basal cell carcinoma manifests as a patchy, erosive lesion that is slightly scaly and has erythematous or flesh-toned attributes, characterized by translucent, rolled borders and tiny erosions, primarily situated on the trunk. Morpheaform (sclerosing) basal cell carcinoma presents as a waxy, scar-like indurated plaque with indistinct margins, generally infiltrative and difficult to define clinically. The pigmented basal cell carcinoma can be confused with melanoma, displaying brown, blue, or black pigmentation alongside pearlescent blemishes. Dermoscopy generally demonstrates arborizing vessels, blue-gray ovoid nests or globules, leaf-like regions, and glistening white structures, facilitating the differentiation of basal cell carcinoma from vascular or melanocytic lesions. BCC histologically consists of clusters of basaloid cells, distinguished by peripheral palisading and cleft formation in the adjacent stroma. Specific histologic subtypes display aggressive characteristics, notably micronodular, infiltrating/morpheaform, and basosquamous basal cell carcinoma, which show heightened invasion and recurrence risk. While metastasis is rare, prompt detection might lead to tissue damage in the impacted area. Unlike SCC, which features scaly, keratotic, and frequently sensitive lesions, BCC manifests as smooth, pearly, and painless lesions. The distinctive characteristics of BCC include pearly translucency, arborizing vasculature, and rolled, ulcerated borders. The clinical and dermoscopic evaluation of the subtype is essential for accurate identification, hence improving treatment planning and reducing recurrence.

**Figure 11:**
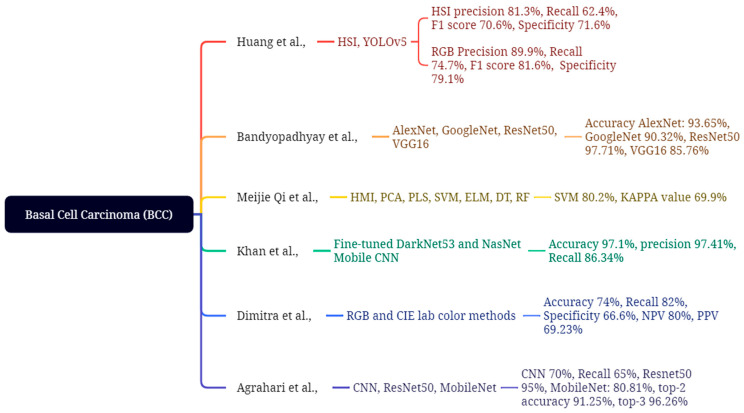
Machine learning application for BCC classification and detection [120,121,122,123,124,125].

Huang et al. evaluated the efficacy of hyperspectral imaging (HSI) in comparison to conventional RGB imaging. The research employed the HSI and YOLOv5 models on 654 training images and 168 validation images, utilizing wavelength ranges of 405–435 nm and 535–555 nm for the detection and analysis of blood heme to extract skin lesions, culminating in the generation of HSI and NBI images, as depicted in Figure 11. The RGB model achieves a precision of 89.9%, a recall of 74.7%, an F1 score of 81.6%, and a specificity of 79.1%. Conversely, the HSI model demonstrates a lower precision of 81.3%, a recall of 62.4%, an F1 score of 70.6%, and a specificity of 71.6%, respectively [120]. In this study, Bandyo-padhyay et al. enhance and diagnose the process for improving basal cell carcinoma (BCC) disease detection by integrating deep learning (DL) and machine learning (ML) models with support vector machine (SVM), AdaBoost, and decision tree (DT) ensemble classifiers, achieving accuracies of 93.65%, 90.44%, and 70.34% for AlexNet; 90.32%, 86.10%, and 68.25% for GoogleNet; 91.71%, 84.96%, and 69.22% for ResNet50; and 85.76%, 83.09%, and 63.02% for VGG16, respectively [121]. This study by Meijie Qi et al. utilizes synthetic RGB data obtained from a hyperspectral microscopic imaging (HMI) cube to classify basal cell carcinoma (BCC). This emphasizes HMI for data collection and visual feature extraction methods, employing color moments of PCA and PLS to process GLCM and LBP for dimensionality reduction. The four classification methods are Extreme Learning Machine (ELM), Support Vector Machine (SVM), Decision Tree (DT), and Random Forest (RF). GLCM and LBP features achieved a maximum accuracy of 66.1 ± 63%, 79.1 ± 60%, 67.1 ± 44%, and 70.6 ± 56%, with corresponding KAPPA values of 49.0 ± 95%, 68.5 ± 95%, 50.5 ± 68%, and 53.3 ± 85%. The SVM achieved optimal performance in BCC classification, attaining an accuracy of 80.2% and a KAPPA score of 69.9% [122].

Khan et al. utilized deep learning to autonomously categorize the multi-class skin lesion issue. The techniques consist of two primary phases: localization and classification. The lesion localization utilizes an innovative convolutional neural network and image fusion techniques. The ISBI2019, ISBI2018, and HAM10000 datasets are utilized to train and assess two models: the fine-tuned DarkNet53 and the fine-tuned NasNet Mobile CNN. The HAM10000 dataset has 10,015 images of diverse skin lesions. The ISBI2018 dataset comprises 10,015 images depicting seven types of skin lesions, whereas the ISIC2019 dataset includes eight unique categories. The classification phase involves the enhancement of two pre-trained models for feature extraction by transfer learning, which are subsequently combined using parallel entropy correlation (PEnC). In the entropy kurto-sis-controlled whale optimizer (EKcWO), selected attributes are classified using SoftMax. The results demonstrate that SoftMax achieved the highest accuracy of 97.1%, a sensitivity of 86.34%, and a precision of 97.41%, respectively [123]. This study by Dimitra et al. examined the diagnosis of malignant cutaneous carcinomas and benign lesions by digital color analysis of photographs. Skin lesions were examined in 46 individuals employing RGB and CIE Lab color methodologies, revealing 11 patients with MCC lesions, 12 with hemangiomas, 12 with BCC, and 11 with cherry angiomas. The results demonstrated an accuracy of 74%, sensitivity of 82%, specificity of 66.6%, negative predictive value (NPV) of 80%, and positive predictive value (PPV) of 69.23% [124]. This study by Agrahari et al. focused on a deep learning framework for the detection of basal cell carcinoma (BCC), employing a fundamental convolutional neural network (CNN) model to understand key deep learning concepts, including convolutional layers, pooling, dropout, and activation functions. ResNet50 utilizes shortcuts to mitigate learning challenges and improve accuracy, especially when implementing a MobileNet model for enhanced precision and efficiency. It utilizes transfer learning, initially pre-trained on the ISIC dataset and later refined on the HAM10000 dataset for skin lesion classification. The collection comprises 10,010 photographs of skin lesions, categorized as follows: 6700 melanocytic nevi, 1113 melanomas, 1099 benign keratoses, 514 basal cell carcinomas (BCCs), 327 actinic keratoses, 142 vascular lesions, and 115 dermatofibromas. The results demonstrate that the fundamental CNN achieved a training accuracy of 70% and a testing accuracy of 65%. Conversely, Res-Net50 attained a training accuracy of 95% and a testing accuracy of 82%. MobileNet achieved an accuracy of 80.81%, with a top-2 accuracy of 91.25% and a top-3 accuracy of 96.26% [125].

### 4.2. Squamous Cell Carcinoma (SCC)

SCC, the second most common keratinocyte malignancy, generally appears in regions of the skin that have experienced prolonged sun exposure, predominantly on the head, neck, ears, lips, and hands. This results from the malignant growth of keratinocytes, usually originating from pre-existing actinic keratoses or Bowen’s disease (in situ SCC). Clinically, SCCs manifest as hardened, hyperkeratotic or crusted, erythematous papules or plaques that may ulcerate and can be unpleasant or uncomfortable, in contrast to the predominantly asymptomatic and smooth BCC. In contrast to their less aggressive biological characteristics, lesions proliferate over a span of weeks to months. Dermoscopy identifies essential features such as white circles, central keratinous masses or crusts, and looping or glomerular vessels on a reddish background, histologically linked to keratin pearls and vascular proliferation. In contrast to BCC, SCC does not exhibit arborizing vasculature or translucency; rather, it displays a rough, scaly, or keratotic surface. The majority of SCCs are responsive to localized treatment; nonetheless, metastasis occurs in 5 to 15 percent of instances, with heightened risk in immunosuppressed patients, lesions situated on the lip or ear, or recurring tumors. The previously listed issues require immediate diagnosis and removal, as it is essential to continuously monitor individuals, especially those with numerous actinic keratoses or a history of non-melanoma skin cancer. In conclusion, SCC is a clinically significant, rapidly proliferating keratotic lesion with the capacity to ulcerate and metastasize, in sharp contrast to BCC, which has slower growth, translucency, and less aggressiveness.

**Figure 12:**
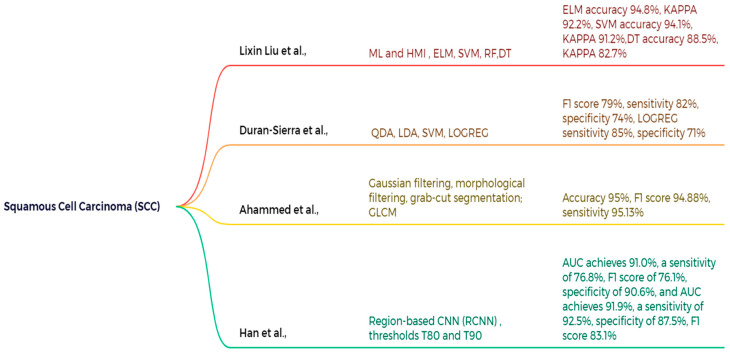
Machine learning application for SCC classification and detection [126,127,128,129].

This study by Lixin Liu et al. examines the stage identification of squamous cell carcinoma (SCC) with machine learning (ML) and human–machine interface (HMI) technologies for skin cancer detection. The spectral data was preprocessed using the second derivative method (SD), first derivative method (FD), multiplicative scatter correction (MSC), principal component analysis (PCA), and partial least squares (PLS), with standard normal variate (SNV) utilized for the staging identification of SCC. The SNV preprocessing model achieves exceptional performance in multiple categories. The model employing RF with SNV preprocessing achieves the highest accuracy and kappa value among all combinations. The accuracy and kappa statistics are as follows: ELM: 94.8 ± 0.9%, 92.2 ± 1.4%; SVM: 94.1 ± 1.5%, 91.2 ± 2.2%; DT: 88.5 ± 1.1%, 82.7 ± 1.7%; RF: 95.2 ± 0.014%, 92.8 ± 2.2% [126]. Duran-Sierra et al. performed clinical imaging validation of maFLIM using a machine learning model for dysplastic and malignant oral lesions in squamous cell carcinoma (SCC) and created a computer-aided diagnosis system applied to endoscopic maFLIM pictures of 23 patients. The research utilized diverse models on maFLIM feature pools, employing 7-fold cross-validation to assess F1-Score, Sensitivity, and Specificity across several classification methods, including quadratic discriminant analysis (QDA), linear discriminant analysis (LDA), support vector machines (SVM), and logistic regression (LOGREG). In spectral feature classification, the SVM model achieved a maximum F1-Score of 79%, with a sensitivity of 82% and a specificity of 74%. LOGREG demonstrated commendable performance, with an F1-Score of 79%, a sensitivity of 85%, and a specificity of 71%. In time-resolved QDA, optimal performance was attained with an F1-score of 83% and a sensitivity of 91%. The SVM demonstrated the lowest performance, recording an F1-score of 73%, a sensitivity of 76%, and a specificity of 68%. An ensemble model that combines features from spectral and time-resolved datasets, employing SVM and QDA, achieves a sensitivity of 94%, specificity of 74%, and an F1 score of 85%, as illustrated in Figure 12 [127].

This article by Ahammed et al. enhances clinical imaging techniques through Gaussian filtering, morphological filtering, and grab-cut segmentation approaches for the identification of affected lesions. The gray level co-occurrence matrix (GLCM) techniques are applied for statistical feature parameters, while the three machine learning algorithms implemented for categorizing SCC skin lesions are SVM, KNN, and DT. The ISIC 2019 and HAM10000 datasets are employed to assess the other two models. The SVM achieves an accuracy of 95%, a precision of 95.13%, and an F1 score of 94.88%, respectively [128]. This study by Han et al. evaluates a deep learning algorithm for the diagnosis of skin cancer. T80 and T90 are thresholds utilized to assess the algorithm’s effectiveness at heightened sensitivity levels. It employs a region-based convolutional neural network (RCNN) to classify and identify 2844 images from 673 patients. The dataset AUC achieves 91.0%, with a sensitivity of 76.8%, an F1 score of 76.1%, and a specificity of 90.6%. Among 80 patients analyzed from 325 photographs, the AUC attained 91.9%, with a sensitivity of 92.5%, specificity of 87.5%, and an F1 score of 83.1%, respectively [129].

### 4.3. Seborrheic Keratosis (SK)

SK is a benign tumor derived from keratinocytes, commonly found in adults of both sexes, especially those with lighter skin, and is more frequently observed in middle-aged and older individuals without gender bias. SKs show clinically as well-defined, adhering, waxy, or verrucous plaques that range in color from light tan to dark brown or black. Harmless and non-premalignant seborrheic keratoses do not advance to melanoma or squamous cell carcinoma and are usually treated for cosmetic or symptomatic purposes. Dermoscopy demonstrates distinctive diagnostic features such as milia-like cysts (white spots), comedo-like orifices (keratin-filled pits), fissures and crevices displaying cerebriform topography, and hairpin arteries encircled by white halos. These characteristics are essential for differentiating seborrheic keratosis from pigmented basal cell carcinoma or melanoma, which typically lack comparable features. Skin keratoses primarily appear on the trunk, face, and extremities; however, an unusual outbreak of multiple lesions, indicative of the Leser-Trélat phenomenon, may imply an underlying internal malignancy and necessitates further investigation only in the presence of accompanying systemic symptoms. A biopsy should be conducted solely on atypical lesions with rapid changes, ulceration, or bleeding. In summary, seborrheic keratoses are benign, adherent lesions with unique dermoscopic features that aid in clinical diagnosis and provide reassurance to the patient regarding their health. It is crucial to recognize them as benign to avert unnecessary removal or concern.

**Figure 13:**
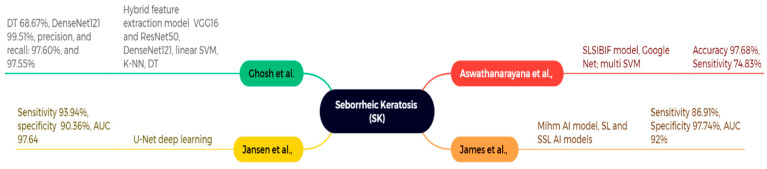
Machine learning application for SK classification and detection [130,131,132,133].

In this study, Aswathanarayana et al. presented an innovative saliency-based level set technique incorporating an improved border indicator function (SLSIBIF) for skin cancer detection. The segmented images were acquired using Google Net, and the effectiveness of their SLSIBIF-MSVM model was assessed in comparison to other methods, including fuzzy K-means with the grasshopper optimization methodology, region-based CNN, and ResNet50, as depicted in Figure 13. Their methodology achieved a classification accuracy of 97.68% and a sensitivity of 74.83% on the ISIC 2017 dataset of 2750 images, including SK [130]. James et al. created the Mihm AI model to improve the identification and categorization of skin lesions related to SK. They have assessed supervised learning (SL) and semi-supervised learning (SSL) artificial intelligence models for the detection and localization of five primary skin lesions, including seborrheic keratosis (SK). This study included various datasets from Pathology Watch, including 2188 supervised whole slide images (WSIs), 5161 inadequately supervised WSIs, and 250 meticulously curated validation WSIs. The findings demonstrate a sensitivity of 86.91%, specificity of 97.74%, and an AUC of 92% for SK, respectively [131].

This study by Jansen et al. distinguishes SK from BD by employing a deep learning algorithm based on the U-Net model to differentiate imaging segmentation of various staining procedures and slide scanners within a robust framework. The datasets utilized include center 0 with 3213 complete slide images, center 1, and center 2. The SK findings indicate an AUC of 97.64, sensitivity of 93.94%, and specificity of 90.36%, respectively [132]. Ghosh et al. utilize a dataset of 3000 images representing nine types of skin cancer lesions, employing a hybrid feature extraction model that combines two pre-trained CNN architectures, VGG16 and ResNet50. Alternative models, including DenseNet121, linear SVM, K-NN, and decision trees, had differing performance levels. The findings revealed the following accuracy: DT 68.67%, DenseNet121 99.51%, with precision and recall at 97.60% and 97.55%, respectively [133].

### 4.4. Actinic Keratosis (AK)

**Figure 14:**
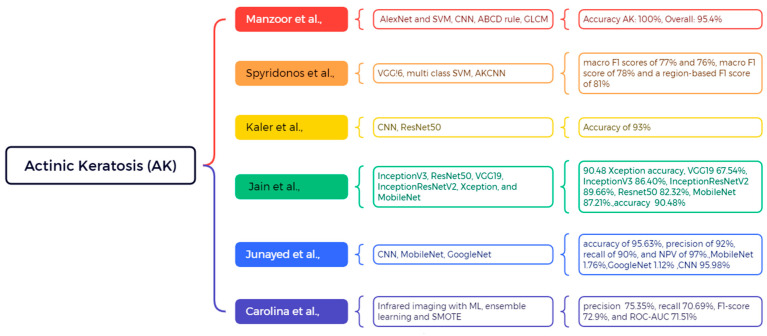
Machine learning application for AK classification and detection [134,135,136,137,138].

AK is a benign lesion that develops on skin exposed to extended UV radiation, commonly located on the face, scalp, forearms, and dorsal hands. AK is a variety of atypical clonal epidermal keratinocytes caused by cumulative UV radiation damage and is presently categorized as a histopathologic in situ SCC. Actinic keratoses are clinically defined as macules, papules, or plaques that exhibit a sandpapery, rough, and scaly texture, which patients frequently detect by touch before visual identification. They may exhibit erythema, hyperkeratosis, or develop cutaneous horns in advanced stages. AK functions as an indicator of cancerization in photodamaged skin regions, characterized by numerous atypical foci, and significantly increases the risk of SCC, with an estimated lifetime transformation rate of approximately 8%; however, the data varies according to cohort and geographic location. The dermoscopic presentation of actinic keratoses on the face, especially in initial stages, often displays the distinctive strawberry pattern, characterized by a reddish pseudonetwork with follicular openings, together with white halos and scales. Due to its malignant traits, actinic keratosis necessitates proactive management using cryotherapy, topical chemotherapeutic agents (including 5-fluorouracil, imiquimod, or diclofenac), or photodynamic therapy, especially in instances of many or confluent lesions. Regular appointments with a dermatologist and rigorous sun protection are crucial for preventing recurrence and development. Actinic keratoses are premalignant, erythematous, scaly papules on sun-damaged skin that should not be overlooked, as they may advance to invasive squamous cell carcinoma as shown in Figure 14. Manzoor et al. aimed to automate a technique for identifying skin disorders, focusing on six common skin malignancies by employing datasets from the ISIC repository and Mendeley. The skin cancer classification utilizes AlexNet and SVM, whereas the proposed methodology integrates segmentation via CNN, the ABCD rule, GLCM, and deep features for feature extraction. The outcome attained an accuracy of 100% in the AK and an overall accuracy of 95.4% [134]. In this study, Carolina et al. utilize infrared imaging techniques alongside machine learning to differentiate skin cancer. The concept utilizes ensemble learning and deep learning algorithms on thermal parameters obtained from thermograms. SMOTE improves the efficacy of classification models. In this scenario, SMOTE generates synthetic samples to balance class distribution and enhance the performance of minority classes, but the lack of SMOTE utilizes the original imbalanced dataset, leading to diminished performance for minority classes. Dataset expansion (DE) outperforms the SCC and AK, with an accuracy of 75.35%, a recall of 70.69%, an F1-score of 72.9%, and a ROC-AUC of 71.51%. The models utilized in SVM and RF attained an accuracy of 75.67%, a sensitivity of 79.07%, and a specificity of 69.67%, respectively [135]. Spyridonos et al. want to create an automated method for identifying actinic keratosis skin lesions affected by cutaneous field cancerization (CFC) using cross-polarized digital images. Feature layers pool3, pool5, and FC7 of VGG16 were employed to train multi-class SVM models. The VGG16 model was assessed utilizing 19,739 AK patches, 43,067 SK/LS patches, and 12,205 healthy skin patches from 46 patients. Pool3 and Pool5 demonstrate superior performance in AK identification, attaining macro F1 scores of 77% and 76%, respectively. The automated algorithm AKCNN achieved a macro F1 score of 78% and a region-based F1 score of 81%, respectively [136]. This study aims to improve the classification of skin disorders by deep learning methods, enabling the early detection of dermoscopic images, as suggested by Kaler et al. The CNN architecture and ResNet-50 are employed on a smartphone linked to the lens as a dermoscopic device for the classification of skin lesions, utilizing 25,000 dermoscopic images. The outcome achieved an accuracy of 93%. In this study, Jain et al. utilized the HAM10000 dataset for multi-class skin cancer classification to assess six different transfer learning models: InceptionV3, ResNet50, VGG19, InceptionResNetV2, Xception, and MobileNet. The attained accuracies were as follows: Xception 90.48%, VGG19 67.54%, InceptionV3 86.40%, InceptionResNetV2 89.66%, ResNet50 82.32%, MobileNet 87.21%. The Xception network achieved the highest accuracy of 90.48% [137]. In this proposed study, Junayed et al. enhance the existing methods by developing a CNN model that exceeds the performance of the pre-trained model. The collection consists of 21 photographs obtained from Dhaka Medical College and 779 images from DermNet, covering four kinds of skin cancer: AK, BCC, MM, and SCC. The AK findings provide an accuracy of 95.63%, precision of 92%, recall of 90%, and a negative predictive value of 97%. In contrast, the CNN model achieves an accuracy of 95.98%, exceeding MobileNet’s by 1.76% and GoogleNet’s performance as referenced in [138]. A comparative assessment of significant studies, encompassing their datasets, techniques, performance indicators, and acknowledged shortcomings, is provided in Table 1.

To beyond mere description and provide practical guidance, machine-learning research on non-melanoma skin lesions must be assessed concerning three translational obstacles: data, validation, and deployment, accompanied by precise reporting metrics. The majority of datasets exhibit imbalance. Research should include class counts and prevalence, present metrics that account for prevalence, and deliver subgroup results categorized by skin tone, anatomical location, and device/modality, while quantifying results with confidence intervals. Random image-level divisions eliminate patient/site leakage and near-duplicate contamination, while rigorous protocols ensure patient- and center-level segregation, external validation across temporal, geographical, and hardware dimensions, and report calibration at a predetermined operating point suitable for screening. Clarification is necessary about preprocessing and augmentation pipelines, hyperparameters, early-stopping thresholds, and safeguards against augmentation leakage; preregistered analytic plans further bolster credibility. Performance typically declines during domain shifts; domain adaptation, device-aware normalization, and uncertainty estimation are little documented, however are crucial for safe deferral and doctor oversight. Saliency maps should be supplemented with stability assessments and systematic error analyses rather than being presented in isolation. Prospective, workflow-oriented assessments incorporating decision-impact outcomes alongside cost-effectiveness analyses and impartial demographic evaluations are the most effective means to illustrate real-world significance. The literature indicates that deep models generally outperform classical baselines; however, heterogeneity and under-reporting hinder cross-study comparability. Adhering to these standards enables definitive comparisons and facilitates more direct pathways to clinical implementation.

## 5. Discussion and Conclusions

Skin cancer, including melanoma and non-melanoma types, represents a significant health issue. Despite progress in treatment modalities for non-melanoma skin cancers, including BCC, SCC, SK, and AK, early identification remains essential for enhancing treatment efficacy. Timely identification of malignant cancers can prevent serious outcomes and reduce recurrence rates. Prompt identification is essential for improving survival rates in skin cancer. Regular dermatological evaluations by healthcare professionals, together with self-examinations, can promote the early identification of skin cancer. Dermoscopy and biopsy are vital tools for diagnosing skin cancer; however, it is as important to educate patients on identifying the warning signs of skin cancer. An important issue is the early detection of skin cancer, especially in areas with limited access to healthcare. Many persons may not identify the early signs, leading in delayed care, which may cause additional issues. Aggressive subtypes, such as morphea form basal cell carcinoma, demonstrate heightened severity and present greater management challenges, particularly when affecting sensitive areas such as the face and neck. Furthermore, while therapies like cryotherapy and surgical procedures are effective, they sometimes require multiple sessions, creating difficulties for patients. BCC is characterized by its indolent growth, local invasiveness, and limited metastatic potential, whereas SCC exhibits a greater propensity for regional invasion, especially in immunocompromised patients. SK is a benign neoplasm lacking malignant potential, yet it possesses clinical importance as a mimic of early cancer. AK is a precancerous disease that may advance to SCC if not addressed. Comprehending these distinctions is crucial for improving precise diagnosis and treatment. Targeted screening of high-risk populations is more efficacious than widespread population-based approaches. Consistent with clinical and dermoscopic assessments, machine-learning diagnostic models (CNNs, SVMs, and QDA) can augment early detection and enhance diagnostic reliability. Ultimately, improving photoprotection, educating the patient, and performing regular self-examinations are crucial in mitigating the disease burden. This document highlights the clinical and technological priorities identified in this review.

Relying on accuracy or AUC for assessing algorithm performance may conceal clinically meaningful differences in diagnostic thresholds. This review examines sensitivity, specificity, accuracy, F1-score, and positive predictive value (PPV) as reported in the current literature. Convolutional Neural Networks (CNNs) displayed sensitivities of 85.895% and specificities of 88.89%, whilst Support Vector Machine (SVM) and Quadratic Discriminant Analysis (QDA) models revealed similar yet somewhat diminished performance. In most cases of threshold tuning, heightened sensitivity was preferred to minimize false negatives and to detect lesions at an early stage of development or subtle abnormalities, particularly in clinical screening applications. The introduction of several complementary indicators will improve comprehension of the model’s reliability and its prospective clinical application. A notable drawback observed in the analyzed studies relates to data, particularly bias present within the dataset. Benchmark datasets like ISIC and HAM10000 primarily comprise fair-skinned individuals, making them inadequate for generalization to darker skin tones or other ethnic groups. The absence of representation may result in the persistent underachievement of specific demographic groups, hence requiring the external validation of data via the acquisition of multi-ethnic data and imagery in authentic clinical settings. Future research will focus on data diversity and the transparent reporting of demographic composition to guarantee equitable access to AI systems in dermatology. Due to the nature of this being a review research rather than an analytical or experimental one, it was impractical to include figures such as confusion matrices or ROC curves. The photographs originate from the original magazines referenced in this article, and copying them without proper attribution would infringe copyright laws. Likewise, heuristic-explainable-AI methodologies such as Gradient-weighted Class Activation Mapping (Grad-CAM) were omitted due to their necessity for access to model weights and internal feature maps, which exceeds the parameters of a literature review. Moreover, a quantitative comparison with dermatologists’ performance could not be directly conducted due to the differing evaluation processes and datasets utilized in the examined research, making uniform benchmarking impracticable. These criteria should be considered essential guidelines for future empirical research focused on assessing AI models in clinical practice. Recent breakthroughs in deep learning architectures, such as ensemble CNNs and ViT networks, have exhibited improved feature representation and greater interpretability. Incorporating these structures with standardized evaluation measures and multicenter validation studies might enhance the connection between research performance and clinical applicability.

To integrate clinical dermatology with bioengineering, it is essential to ensure the seamless operation of sensing, computation, and workflow. Advancements in imaging technology, including polarized dermoscopy, smartphone-compatible CMOS modules, multispectral and hyperspectral cameras, and depth-resolving modalities, enable the capture of morphological and sub-surface optical signals crucial for the early detection of keratinocytic lesions. Signal processing and spectrum analysis including flat-field correction, illumination normalization, spectral unmixing, and feature fusion across RGB, dermoscopy, and hyperspectral imaging provide machine learning with dependable inputs for further use. Robust pipelines emphasize domain-appropriate preprocessing, management of class imbalance, and models that integrate high sensitivity with effective calibration and reliable uncertainty estimates suitable for triage. Systems engineering enhances the connection between the bench and the bedside by employing edge or near-edge inference to reduce latency and ensure privacy, standardized data formats and metadata, and incorporating a human-in-the-loop review that provides doctors with explanations and failure alerts. When evaluated through patient- and site-specific divisions, external assessments, and prevalence-sensitive metrics, these integrated sensor-to-AI-to-workflow systems translate bioengineering advancements into clinically relevant functions such as risk-stratified triage, margin evaluation, and longitudinal monitoring, while pinpointing domains where additional initiatives—device standardization, spectral benchmarks, and prospective, workflow-integrated trials—are most likely to improve outcomes and guarantee equitable access.

The initiative aims to enhance diagnostic technologies, incorporating non-invasive techniques such as AI-driven imaging, dermoscopy, and genetic testing, to enable earlier cancer detection. Research into personalized medicine, especially the targeting of specific genetic anomalies in skin cancer, may produce more effective therapies. Furthermore, public health activities focused on teaching individuals about the dangers of sun exposure and the importance of sunscreen use would be crucial in prevention efforts. In conclusion, despite significant progress in the treatment and management of skin cancer, early detection and public education are essential for reducing its effects. Continued research and awareness efforts will produce enhanced outcomes and safeguard lives.

This study offers a thorough examination of the epidemiology, screening, clinical aspects, and machine learning techniques related to common non-melanoma skin cancers, such as BCC, SCC, SK, and AK; nevertheless, numerous limitations must be acknowledged. The review was predominantly limited to studies sourced from open-access databases and benchmark image repositories, including ISIC and HAM10000. As a result, important findings published in non-English or subscription-only publications may not have been documented. The performance comparison of several ML and DL models was conducted using results from heterogeneous research with differing dataset sizes, imaging modalities, preprocessing techniques, and validation methods; hence, direct cross-study comparisons are inherently biased. This study was neither a meta-analytic statistical synthesis nor an experimental validation, making the findings on diagnostic accuracy interpretative rather than confirmatory. Furthermore, although the evaluated machine learning models exhibited potential diagnostic efficacy, their clinical applicability is limited by insufficient dataset diversity, potential class imbalance, and the lack of real-world, multi-ethnic validation cohorts. The swift advancement of technological innovation and the evolution of AI models and architectures indicates that emerging models and multimodal diagnostic tools may exceed those previously considered, requiring updates to ensure their usefulness in clinical settings. This analysis underscores the critical importance of early identification in enhancing survival rates and treatment effectiveness for melanoma and non-melanoma skin malignancies. Machine learning and artificial intelligence have considerable potential to improve diagnostic accuracy using non-invasive image analysis. However, these methods cannot be verified with limited datasets and necessitate comprehensive standardized and heterogeneous datasets for implementation in real clinical practice. Informing the public about sun protection and the need of routine dermatological examinations is crucial for prevention and early detection. The incorporation of AI-driven diagnostics and public health programs offers a chance to diminish global skin cancer rates. This review enhances the theoretical understanding of how computational models interpret and categorize dermatological features. The work juxtaposes various machine learning and deep learning methodologies to elucidate theoretical insights into feature extraction, non-linear decision boundaries, and hierarchical pattern learning in biomedical imaging. Convolutional Neural Networks (CNNs) exhibit enhanced efficacy in hierarchical spatial representation when integrated with Support Vector Machines (SVM) and Quadratic Discriminant Analysis (QDA), whereas SVM and QDA provide critical perspectives on linear separability and probabilistic decision modeling. The conceptual framework connecting data classification theory and biological morphology is further augmented by the use of clinical picture interpretation and machine learning principles. Future AI-driven healthcare systems can be structured to integrate interpretability, computational efficiency, and clinical application by using these theoretical implications to create hybrid diagnostic models. This research demonstrates that the implementation of machine learning and deep learning models has significantly enhanced the accuracy and efficiency of skin cancer diagnosis. Studies utilizing benchmark data like ISIC and HAM10000 demonstrated diagnostic accuracy over 90 percent, particularly for non-melanoma lesions, including BCC, SCC, SK, and AK. The CNN-based models exhibited superior performance in automatic image classification, while the SVM and QDA provided valuable interpretability for clinical decision support. Despite these achievements, the findings reveal existing limitations in dataset diversity, an imbalance among lesion types, and the imperative for extensive multicenter validation. The paper emphasizes the enhanced importance of AI-driven diagnostic tools in facilitating early disease detection, optimizing clinical workflows, and improving patient outcomes in dermatological oncology.

## Figures and Tables

**Table 1:** Summary of Reviewed Studies on Machine Learning Approaches for Skin Cancer Detection.

Study (Year)	Dataset/Sample Size	Methodology/Model	Lesion Types	Key Findings	Reported Limitations
Huang et al., 2023 [120]	HSI & RGB images; 654 train + 168 val (non-ISIC)	YOLOv5 (HSI vs. RGB)	BCC	RGB: P 89.9%, R 74.7%, F1 81.6%, Spec 79.1; HSI: P 81.3%, R 62.4%, F1 70.6%, Spec 71.6	Limited spectral window; lower HSI metrics vs. RGB; modest sample size
Bandyopadhyay et al., 2022 [121]	Dataset not specified in manuscript text (general DL/ML ensemble summary)	DL backbones (AlexNet/GoogLeNet/ResNet50/VGG16) + SVM/AdaBoost/DT	BCC, SCC	Accuracies up to 93.65% (AlexNet + SVM), with model-wise results as reported	Computational complexity; no real-time clinical validation noted
Qi et al., 2023 [122]	Hyperspectral microscopic imaging (synthetic RGB); classical ML	PCA/PLS + GLCM/LBP → ELM/SVM/DT/RF	BCC	SVM best: Accuracy 80.2%, κ = 0.699 (others lower)	Small dataset; synthetic-to-real domain gap
Khan et al., 2021 [123]	ISIC2018/ISIC2019/HAM10000 (multi-dataset)	Fine-tuned DarkNet53 & NasNet; fusion + SoftMax	BCC, SCC, SK, AK (+others)	Accuracy 97.1%, Sensitivity 86.34%, Precision 97.41%	Class imbalance; high compute; multi-dataset harmonization
Duran-Sierra et al., 2021 [127]	maFLIM endoscopic imaging (23 pts)	QDA/LDA/SVM/LOGREG on spectral & time-resolved features; ensemble (SVM + QDA)	SCC (oral dysplastic/malignant vs. healthy)	Time-resolved QDA: F1 83%, Sens 91%; Ensemble: F1 85%, Sens 94%, Spec 74% (best overall)	Small, single-center; external validation lacking
Ahammed et al., 2022 [128]	ISIC2019/HAM10000	SVM/KNN/DT on GLCM features	SCC	SVM: Accuracy 95%, Precision 95.13%, F1 94.88%	Hand-crafted features; limited external validation
Aswathanarayana et al., 2023 [130]	ISIC 2017 (*n* = 2750)	GoogleNet features + SLSIBIF-MSVM	SK (with other ISIC’17 classes)	Accuracy 97.68%, Sensitivity 74.83%	Moderate recall for some classes; per-class counts limited
Jansen et al., 2022 [132] (replaces unverified “James/Mihm AI” row)	Multi-center WSIs (center 0: 3213 slides; centers 1 & 2 also used)	U-Net-based DL segmentation/classification	SK vs. Bowen’s disease (BD)	AUC 97.64, Sensitivity 93.94%, Specificity 90.36%	Scanner/stain variability; domain shift possible
Manzoor et al., 2022 [134]	ISIC + Mendeley (>5000 images)	AlexNet features + SVM; segmentation + ABCD + GLCM	AK (+other classes)	AK accuracy 100%; overall 95.4%	Possible overfitting; AK subset size considerations
Spyridonos et al., 2023 [136]	Cross-polarized clinical images; patch-level datasets (AK/SK/healthy)	VGG16 feature layers (pool3/pool5/FC7) + multi-class SVM; AKCNN system	AK, SK, Healthy	Macro F1 = 78%, Region-based F1 = 81% (note: F1, not AUC)	Class imbalance; limited external validation

## Data Availability

No new data were created or analyzed in this study.
